# Activation of Hypoxia Inducible Factor 1 Is a General Phenomenon in Infections with Human Pathogens

**DOI:** 10.1371/journal.pone.0011576

**Published:** 2010-07-14

**Authors:** Nadine Werth, Christiane Beerlage, Christian Rosenberger, Amir S. Yazdi, Markus Edelmann, Amro Amr, Wanja Bernhardt, Christof von Eiff, Karsten Becker, Andrea Schäfer, Andreas Peschel, Volkhard A. J. Kempf

**Affiliations:** 1 Institut für Medizinische Mikrobiologie und Hygiene, Universitätsklinikum Tübingen, Eberhard-Karls-Universität, Tübingen, Germany; 2 Institut für Medizinische Mikrobiologie und Krankenhaushygiene, Klinikum der Johann Wolfgang Goethe-Universität, Frankfurt am Main, Germany; 3 Medizinische Klinik mit Schwerpunkt Nephrologie und Internistische Intensivmedizin, Charité, Berlin, Germany; 4 Département de Biochimie, Université de Lausanne, Epalinges, Switzerland; 5 Universitäts-Hautklinik, Eberhard-Karls-Universität, Tübingen, Germany; 6 Medizinische Klinik 4 – Nephrologie und Hypertensiologie, Friedrich-Alexander-Universität Erlangen-Nürnberg, Erlangen, Germany; 7 Institut für Medizinische Mikrobiologie, Universitätsklinikum Münster, Münster, Germany; Instituto Butantan, Brazil

## Abstract

**Background:**

Hypoxia inducible factor (HIF)-1 is the key transcriptional factor involved in the adaptation process of cells and organisms to hypoxia. Recent findings suggest that HIF-1 plays also a crucial role in inflammatory and infectious diseases.

**Methodology/Principal Findings:**

Using patient skin biopsies, cell culture and murine infection models, HIF-1 activation was determined by immunohistochemistry, immunoblotting and reporter gene assays and was linked to cellular oxygen consumption. The course of a *S. aureus* peritonitis was determined upon pharmacological HIF-1 inhibition. Activation of HIF-1 was detectable (i) in all *ex vivo* in biopsies of patients suffering from skin infections, (ii) *in vitro* using cell culture infection models and (iii) *in vivo* using murine intravenous and peritoneal *S. aureus* infection models. HIF-1 activation by human pathogens was induced by oxygen-dependent mechanisms. Small colony variants (SCVs) of *S. aureus* known to cause chronic infections did not result in cellular hypoxia nor in HIF-1 activation. Pharmaceutical inhibition of HIF-1 activation resulted in increased survival rates of mice suffering from a *S. aureus* peritonitis.

**Conclusions/Significance:**

Activation of HIF-1 is a general phenomenon in infections with human pathogenic bacteria, viruses, fungi and protozoa. HIF-1-regulated pathways might be an attractive target to modulate the course of life-threatening infections.

## Introduction

Mammalian cells adapt to oxygen deprivation by the activation of *hypoxia inducible factor* (HIF)-1, the key transcription factor during hypoxia. Subsequently, the expression of hypoxia-inducible genes involved in angiogenesis [e.g., *vascular endothelial growth factor* (VEGF)], glycolysis [e.g., hexokinase (HK)], proliferation and survival [e.g., adrenomedullin (ADM)] and erythropoiesis (e.g., erythropoietin) is transcriptionally regulated [1], [2]. The heterodimeric transcription factor HIF-1 is composed of the two subunits HIF-1α and HIF-1β. While HIF-1β is constantly present in the nucleus, HIF-1α levels are affected by changes in the cellular oxygen partial pressure (pO_2_). The key mechanism involved in HIF-1 activation has been identified to require inhibition of the enzymatic activity of “prolyl hydroxylase domain”-containing proteins (PHDs) during hypoxia. PHDs mediate hydroxylation of the prolyl residues Pro402 and Pro564 of the HIF-1α subunit which results in the binding to the von-Hippel-Lindau protein and subsequent proteasomal degradation under normoxic conditions. In contrast, hypoxia results in PHD-inhibition and subsequent stabilization of HIF-1α, binding of the HIF-1 heterodimer to promoter regions of hypoxia-inducible genes and corresponding gene induction [2]. Iron deprivation [induced by iron chelating compounds, e.g., desferrioxamine (DFO)] has been found an alternative strategy of HIF-1 activation [3]. The molecular explanation for this phenomenon is that PHDs contain iron as an essential cofactor for their enzymatic activity; therefore, iron chelation inhibits PHD activity resulting in the subsequent activation of HIF-1 [4].

A constantly growing body of evidence suggests that HIF-1 plays a novel and important role in infectious and inflammatory diseases [5]. HIF-1 is essential for the bactericidal capacity of phagocytes and for controlling systemic spread of bacteria in mice [5], [6]. Interestingly, HIF-1 activation occurs during bacterial infections with the angiogenic bacterium *Bartonella henselae* (causing the vasculoproliferative disorder bacillary angiomatosis) [7] and this phenomenon is linked with the expression of the *Bartonella* adhesin A [8]–[10]. In infections with humanpathogenic *Enterobacteriaceae*, HIF-1 activation is the result of iron-competition between bacteria and host cells caused by secreted bacterial siderophores; here, HIF-1 plays an important role in the defense of *Yersinia enterocolitica* infections [11]. Using keratinocytes, it was recently demonstrated that HIF-1 activation results in the expression of cathelicidin, an antimicrobial peptide mediating protection against gram positive group A *streptococci* [6]. LPS from gram negative bacteria can trigger HIF-1 activation in macrophages [12], [13] and this HIF-1 activation is crucial for the development of a LPS triggered sepsis [14], [15]. Interestingly, LPS-dependent HIF-1 activation does not occur using epithelial or endothelial cell-based infection models [9]–[11].

Although several aspects have been analyzed in the last years, the exact role of HIF-1 in the course of infection with human pathogens remains widely unclear. Therefore, we investigated the activation of HIF-1 in infections with human pathogens more generally using human skin biopsies, cell culture techniques and by employing *Staphylococcus aureus* infection models. Our data reveal that HIF-1 might play an important and previously underestimated role in many infectious diseases.

## Results

### Activation of HIF-1 by human pathogens is a general phenomenon in infections

**Figure 1:**
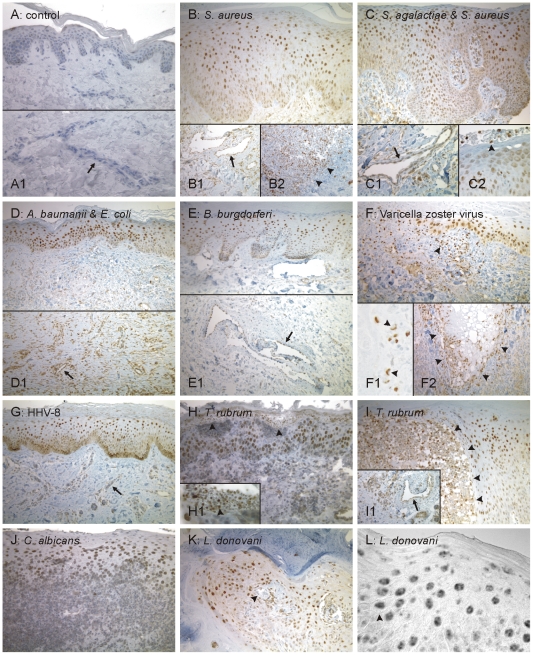
Detection of HIF-1 activation in human skin biopsies of patients suffering from cutaneous infections by immunohistochemistry. (A) In uninfected skin, rare and faint nuclear HIF-1α staining occurred in the epidermis whereas the dermis was negative for HIF-1α (A1). (B to L) In all cutaneous infections, a strong nuclear HIF-1α signal was detected in keratinocytes, predominantly of the spinal cell layer (examplified in L). Moreover, endothelial cells of dermal capillaries frequently stained positive (arrows in B1, C1, D1, E1, G, I1). Arrowheads: (B2) border zone of the positive abscess; (C2): positive neutrophils; (F): positive dermal lympho-histiocytic infiltrates; (F1): large positive nuclei of a multi-nuclear giant cell; (F2): skin lesion surrounded by a HIF-1 positive inflammatory infiltrate; (H): positive sub-corneal neutrophils (higher power in H1); (I): positive intra-epidermal infiltrate; (K): intradermal nodules containing leishmaniae. (A) Control: uninfected sample; (B–E): bioptic samples of patients suffering from infections with humanpathogenic bacteria [(B) *S. aureus*, (C) *S. agalactiae* and *S. aureus*, (D) *A. baumanii* and *E. coli* and (E) *B. burgdorferi*], (F, G) with humanpathogenic Herpes-viruses [(F) Varicella zoster virus (VZV) and (G) Human Herpes Virus-8 (HHV 8)], (H–J) with humanpathogenic fungi [(H+I) *T. rubrum* and (J) *C. albicans*] or (K, L) with the protozoic pathogen *L. donovani*. Magnifications: all 250×, except for A1, B1, B2, C1, D1, E1, F2, I1 (all 400×), and C2, F1, H1, L (all 1,000×).

First, we analyzed *ex vivo* whether pathogen-triggered HIF-1 activation is detectable in biopsy samples of patients suffering from various bacterial, viral, fungal or parasitic skin infections. For this purpose, paraffin-embedded patient skin biopsies were collected according to the following strict inclusion criteria: (a) a clinically suspected infectious process, (b) the histopathological diagnosis of an infection (no malignancy, no autoimmunological disease) and (c) a microbiologically positive laboratory result confirming the presence of specific pathogens in the lesions. In total, four samples of bacterial skin infections, two samples of viral skin infections, three samples of fungal skin infections and two samples of protozoic skin infections were investigated by HIF-1α-specific immunohistochemistry (**Fig. 1**). In control samples (healthy patients, bioptic sample taken for other reasons, **Fig. 1A**), a rare and faint nuclear HIF-1α staining occurred in the epidermis whereas the dermis was negative for HIF-1α. In contrast, in all cutaneous infections [here: infections with *S. aureus* (**Fig. 1B**), coinfection with *S. agalactiae* and *S. aureus* (**Fig. 1C**), coinfection with *Acinetobacter baumanii* and *E. coli* (**Fig. 1D**), infections with *Borellia burgdorferi* (**Fig. 1E**), Varicella zoster virus (**Fig. 1F**), Human Herpes Virus-8 (**Fig. 1G**), *Tinea rubrum* (**Fig. 1H,I**), *C. albicans* (**Fig. 1J**) and *Leishmania donovani* (**Fig. 1K,L**)] a strong nuclear HIF-1α signal was detected in keratinocytes (predominantly of the spinal cell layer), dermal capillaries, neutrophils, dermal lymphocytes and macrophages and sub-corneal neutrophils (for details please see legend of **Fig. 1**).

**Figure 2 pone-0011576-g002:**
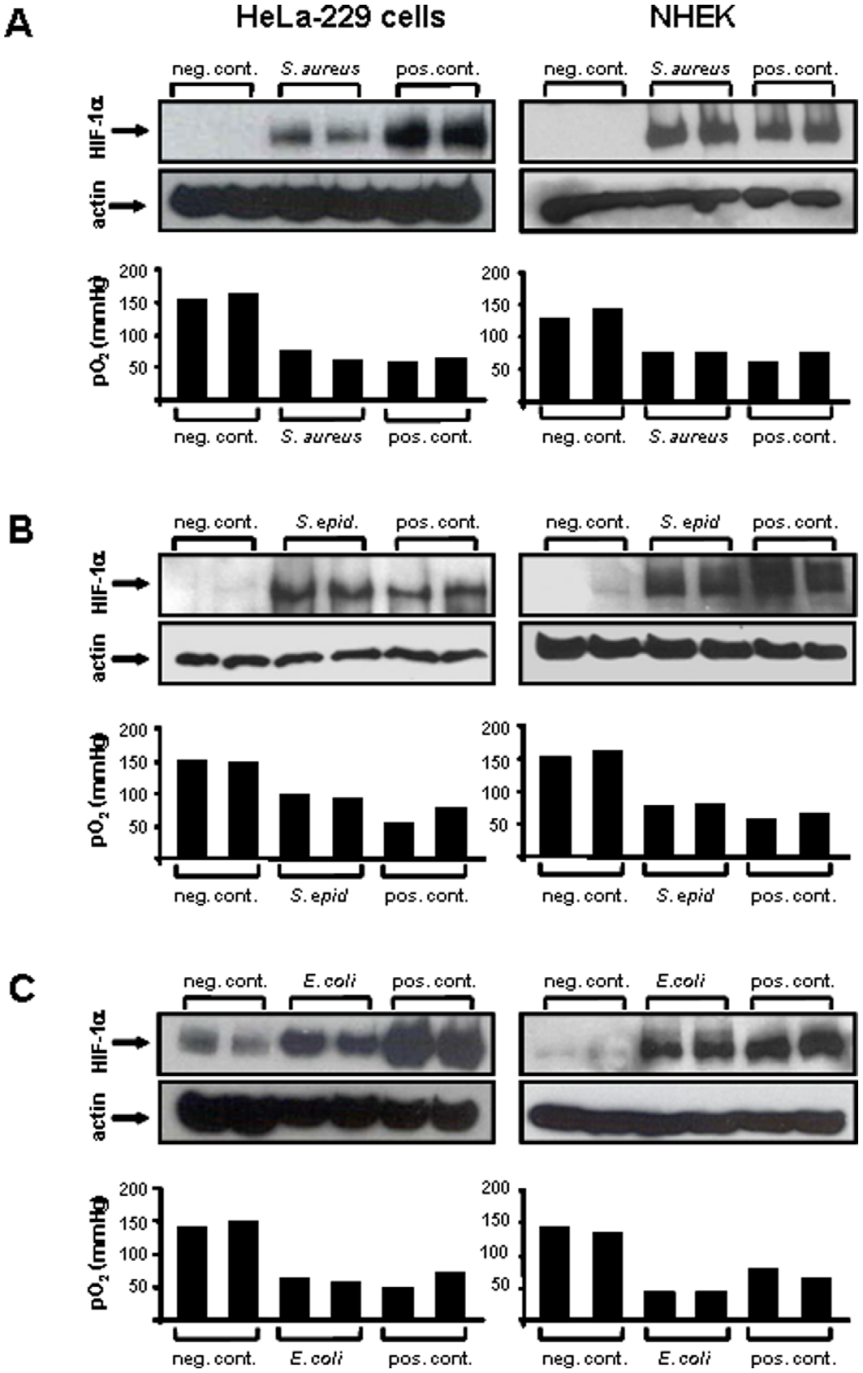
Oxygen-dependent HIF-1 activation in HeLa-229 cells and NHEKs (Normal Human Epidermal Keratinocytes) by bacterial and fungal pathogens. Four to six hours upon infection, HIF-1 activation was determined by Western blotting (loading control: actin). pO_2_ levels were quantified in the medium of control and infected cells (A: *S. aureus* ATCC 33592, MOI 20, infection time: six hours; B: *S. epidermidis* ATCC 12228, MOI 20, infection time: six hours; C: *E. coli* ATCC 25992, MOI 10, infection time: four hours). Negative control: uninfected cells, positive control: hypoxia or DFO (200 µmol/L).

Activation of HIF-1 in host cells upon infections with *B. henselae* and several members of the family of *Enterobacteriaceae* is triggered by oxygen-dependent and -independent mechanisms [7], [11]. Now, we were interested whether and by which mechanisms various human pathogens lead to the activation of HIF-1 in host cells. For this purpose, we infected HeLa-229 cells and NHEKs (Normal Human Epidermal Keratinocytes) with a collection of bacterial pathogen reference strains (*S. aureus* ATCC 33592, *S. epidermidi*s ATCC 12228, *E. coli* ATCC 25922, *P. aeruginosa* ATCC 27853, *S. agalactiae* SK 43) and with the fungal pathogen *C. albicans* ATCC 90028. In all these *in vitro* infection models, a robust activation of HIF-1 was detectable via HIF-1α immunoblotting (**Fig. 2** **,** **3**) and congruent results were obtained via immunohistochemistry using Hela-229 cells grown on coverslips (**Fig. S1**, not all data shown). HIF-1 activation was correlated with increased oxygen consumption of the respective infected host cells strongly arguing for oxygen-dependent HIF-1 activation mechanisms. This suggestion is confirmed by the observation that HIF-1 activation by *S. aureus*, *P. aeruginosa* and *E. coli* was overcome by culturing infected cells in gas-permeable dishes (data not shown). Accordingly, induction of the HIF-1-regulated VEGF was detected in bacterial infections with *S. aureus*, *P. aeruginosa* and *E. coli* by VEGF mRNA induction whereas HIF-1α mRNA transcript levels itself again appeared unaffected (**Fig. S2**). Infections with *S. pyogenes* did not lead to oxygen consumption nor HIF-1 activation (data not shown).

**Figure 3 pone-0011576-g003:**
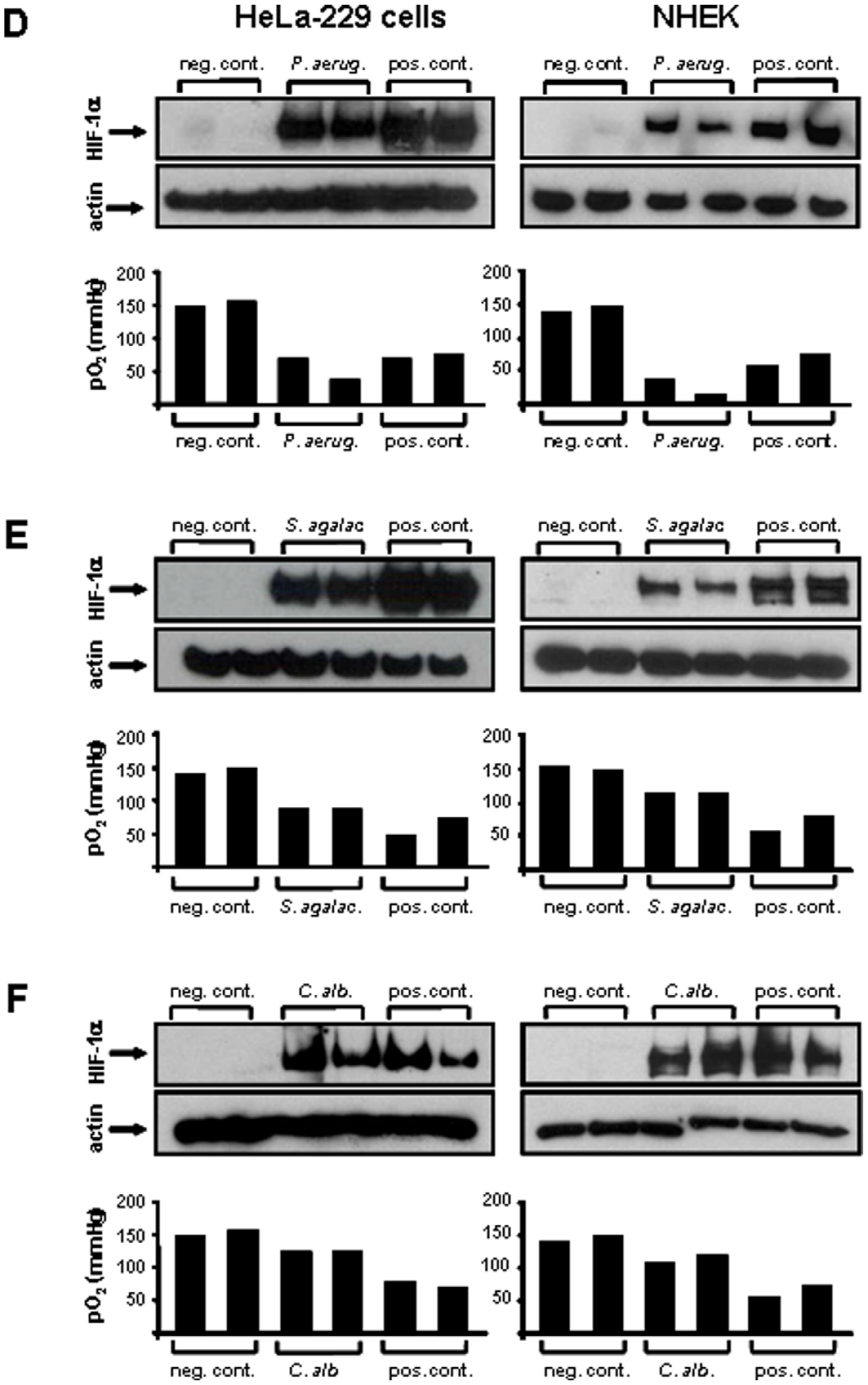
Oxygen-dependent HIF-1 activation in HeLa-229 cells and NHEKs (Normal Human Epidermal Keratinocytes) by bacterial and fungal pathogens. Four to six hours upon infection, HIF-1 activation was determined by Western blotting (loading control: actin). pO_2_ levels were quantified in the medium of control and infected cells (D: *P. aeruginosa* ATCC 27853, MOI 10, infection time: four hours; E: *S. agalactiae* SK 43, MOI 200, infection time: five hours; F: *C. albicans* ATCC 90028, MOI 20, infection time: six hours). Negative control: uninfected cells, positive control: hypoxia or DFO (200 µmol/L).

### Infection of host cells with *S. aureus* results in HIF-1-regulated gene programming

Infection of host cells with *B. henselae* or *Enterobacteriaceae* (*Y. enterocolitica, Enterobacter aerogenes, Salmonella enterica*) results in the activation of HIF-1 and a subsequent HIF-1-dependent angiogenic gene programming *in vitro* and *in vivo* [7], [9]–[11]. As HIF-1 activation was also detectable in infections with *S. aureus* (see above), a clinically most important pathogen, we focussed on this bacterium to elucidate which mechanisms and which biological consequences underlie this process.

**Figure 4:**
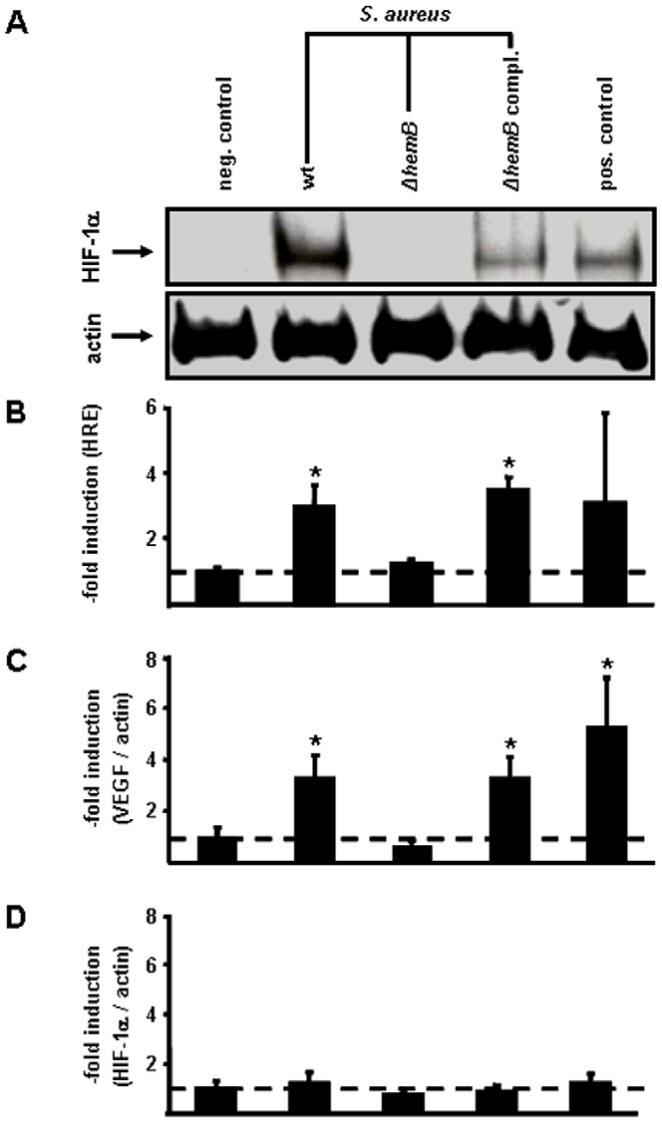
Induction of HIF-1-dependent gene programming in HeLa-229 cells by different *S. aureus* strains. Six hours upon infection, HIF-1 activation was analyzed in (A) Western blots (loading control: actin) and by determining (B) the induction of the HIF-1-dependent luciferase activity. HeLa-229 cells were transfected with a 4xHRE-luc reporter construct 24 hours before infection (triplicate means given; for details see Material and Methods). Induction was determined by chemiluminescence. Bacterial induced (C) VEGF and (D) HIF-1α transcription. Total mRNA was prepared six hours upon infection, transcribed into cDNA and quantified by real-time Light-Cycler-PCR (ratio: VEGF/actin or HIF-1α/actin transcripts; triplicate means given). Negative control: uninfected cells; *S. aureus* wt (8325-4); *S. aureus* Δ*hemB* (*S. aureus ermB*Ω*hemB* 8325-4, SCV); *S. aureus* Δ*hemB*, *hemB*-complemented (*S. aureus* pCX19Ω*hemB* 8325-4, complemented SCV mutant) [16]; positive control: DFO (200 µmol/L). Multiplicity of infection (MOI): 20. * significant difference to control cells (P<0.05).

First, we infected HeLa-229 cells with a well characterized *S. aureus* laboratory strain (8325-4). Such infection led to a robust HIF-1 activation and HIF-1 dependent VEGF induction shown by HIF-1α-immunoblotting, HIF-1-dependent luciferase reporter assays and quantitative PCR analysis (**Fig. 4A, B, C**). HIF-1α mRNA transcript levels itself were unaffected **(** **Fig. 4D** **)** excluding direct effects of *S. aureus* on HIF-1 transcription but suggesting oxygen-dependent posttranslational mechanisms of HIF-1 activation.

**Figure 5:**
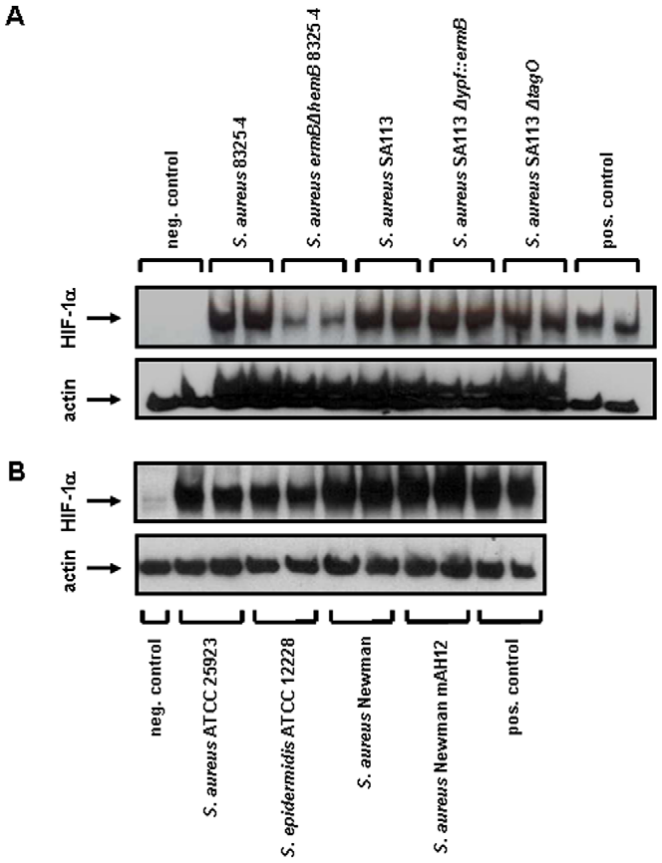
Induction of HIF-1 activation in HeLa-229 cells by different *S. aureus* strains defective in the expression of cell wall components. The HIF-1α protein was analyzed in Western blots six hours upon infection (loading control: actin) of HeLa 229 cells with the following bacterial strains: (A) *S. aureus* 8325-4 (wt), *S. aureus ermB*Ω*hemB* 8325-4 (SCV), *S. aureus* SA113 (wt), *S. aureus* SA113 Δ*ypf::ermB* (reduced in lipoteichoic acid synthesis), *S. aureus* SA113 Δ*tagO* (defective in producing wall teichoic acid); (B) *S. aureus* ATCC 25923 (wt), *S. epidermidis* ATCC 12228 (wt), *S. aureus* Newman (wt), *S. aureus* Newman mAH12 (defective in producing the extracellular adherence protein EAP). Negative control: uninfected cells, positive control: DFO (200 µM). Multiplicity of infection (MOI): 20.

Next, we introduced a genetically defined small colony variant of *S. aureus* (*S. aureus* Δ*hemB*) and the *S. aureus* Δ*hemB hemB-*complemented strain (*S. aureus pCX19ΩhemB*) in our experiments. *S. aureus* Δ*hemB* shows a small-colony phenotype and is auxotrophic for the synthesis of hemin [16]. Therefore, as hemin is essential for the biosynthesis of cytochromes, *S. aureus* Δ*hemB* is deficient in aerobic metabolism [17]. Interestingly, *S. aureus* Δ*hemB* was neither capable in activating HIF-1 nor in VEGF induction whereas the complemented strain was fully restored in its biological effects (oxygen consumption, HIF-1 activation and VEGF induction) suggesting oxygen-dependent mechanisms in the activation of HIF-1 by *S. aureus*. Furthermore, *S. aureus* strains defective in certain cell wall components known to be important for pathogenicity [e.g., lipoteichoic acid [18], wall teichoic acid [19], extracellular adherence protein [20]] were not impaired in their ability of inducing HIF-1 activation (**Fig. 5**).

**Figure 6:**
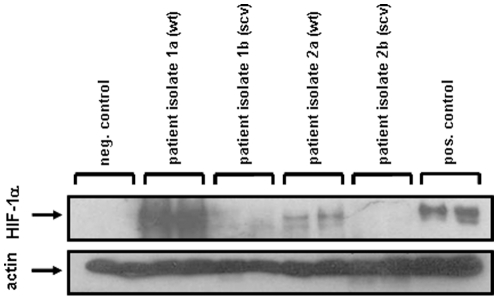
Induction of HIF-1 activation in HeLa-229 cells by *S. aureus* patient isolates (wild-type and the respective small colony variants). The HIF-1α protein was analyzed in Western blots six hours upon infection (loading control: actin). The following patient isolates were used: *S. aureus* patient isolate 1 (wt; *S. aureus* A22223 I), *S. aureus* patient isolate 2 (clinical SCV; *S. aureus* A22223 II), *S. aureus* patient isolate 3 (wt; *S. aureus* OM 1a), *S. aureus* patient isolate 4 (clinical SCV; *S. aureus* OM 1b). Negative control: uninfected cells, positive control: hypoxia. Multiplicity of infection (MOI): 20.

To exclude that the above described phenomenon might be restricted to this particular *S. aureus* laboratory strain, we employed several other *S. aureus* small colony variants (clinical SCVs) originating from patients suffering from chronic infections and their respective parental wild-type strain (**Fig. 6**, not all data shown). Data clearly revealed that only wild-type but not clinical SCV bacteria lead to the activation of HIF-1 underlining the general aspect of our observations. Further analysis revealed that the clinical SCV *S. aureus* OM 1b is auxotrophic for menadione [21] and *S. aureus* A22223 II is unable to synthesize hemin [22]. Both auxotrophies are linked to the activity of the electron transport chain and affect thereby the aerobic metabolism.

**Figure 7:**
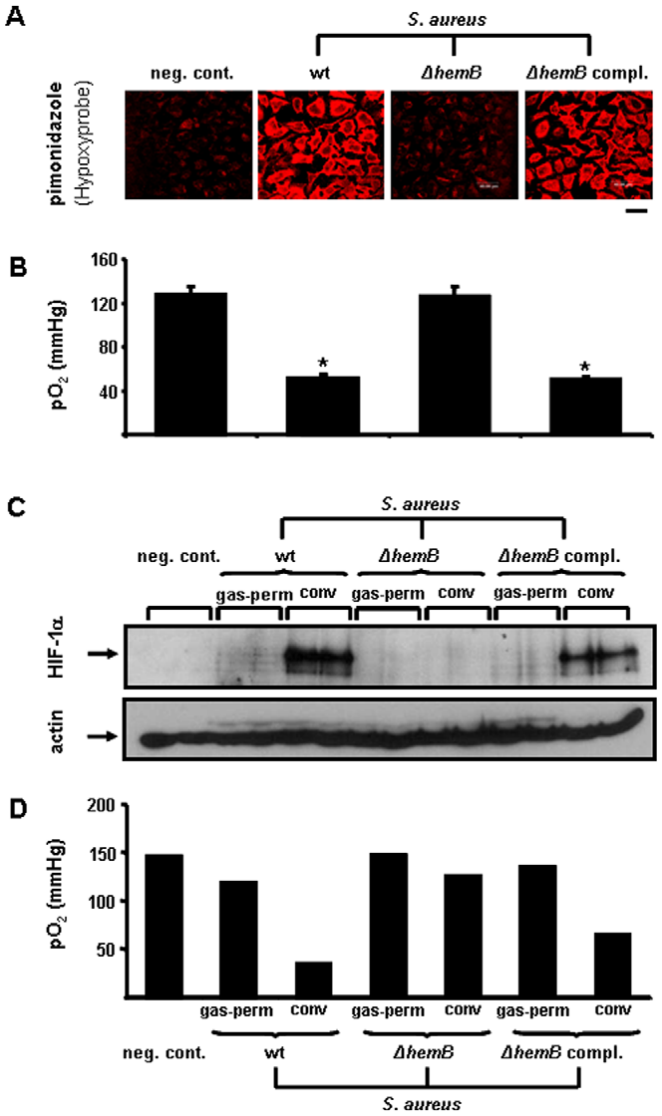
Cellular oxygen consumption and oxygen-dependent HIF-1 activation in HeLa-229 cells infected with different *S. aureus* strains. (A) Control and infected HeLa-229 cells were incubated with the hypoxic marker pimonidazole hydrochloride which was visualized by using specific Cy3 labelled antibodies (for details see Materials and Methods). Scale bare: 40 µm. (B) pO_2_ levels were quantified in the medium of control and infected cells (triplicate means given). (C) Hela-229 cells were incubated in gas permeable (gas-perm) and conventional (conv) culture dishes and HIF 1α protein was analyzed in Western blots (loading control: actin). (D) pO_2_ levels in the medium of control or *S. aureus* infected HeLa-229 cells (means of doubles). Negative control: uninfected cells; *S. aureus* wt (8325-4); *S. aureus* Δ*hemB* (*S. aureus ermB*Ω*hemB* 8325-4, SCV); *S. aureus* Δ*hemB*, *hemB*-complemented (*S. aureus* pCX19Ω*hemB* 8325-4, complemented SCV mutant). Multiplicity of infection (MOI): 20. * significant difference to control cells (P<0.05).

To prove whether HIF-1 activation by *S. aureus* is an oxygen-dependent process, we determined cellular hypoxia upon a bacterial infection using a hypoxia-sensitive dye and by quantification of the dissolved O_2_ concentration in cell culture supernatants. In fact, cellular hypoxia occured in infections with *S. aureus* 8325-4 and the *S. aureus* Δ*hemB hemB-*complemented strain but not when cells were infected with *S. aureus* Δ*hemB*. Here, a significantly increased oxygen consumption in cells infected with *S. aureus* 8325-4 and the *S. aureus* Δ*hemB hemB-*complemented strain was detectable (**Fig. 7A, B**). To analyze whether such HIF-1 activation by *S. aureus* might be overcome by oxygen, an infection model using conventional versus gas-permeable cell culture dishes was introduced allowing to investigate oxygen-dependent and -independent mechanisms of HIF-1 activation. As expected, activation of HIF-1 by *S. aureus* 8325-4 and the *S. aureus* Δ*hemB hemB-*complemented strain did not occur in gas-permeable culture dishes and this correlated clearly with the oxygen partial pressure in the respective cell culture supernatants. For *S. aureus* Δ*hemB* no HIF-1 activation nor oxygen consumption was detectable in infected host cells (Fig. **7C, D**). Taken together, these results show that (i) *S. aureus* wildt-ype but not *S. aureus* SCV induces a HIF-1 regulated gene programming in host cells and that (ii) HIF-1 activation is in fact an oxygen dependent process.

**Figure 8:**
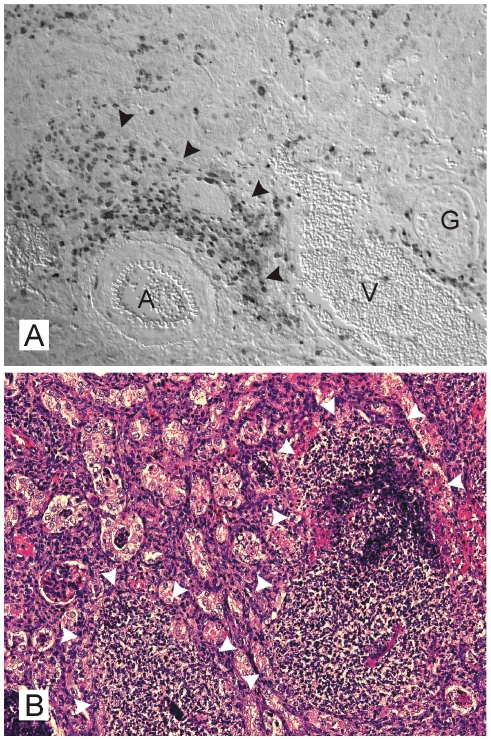
HIF-1 activation in kidneys of mice infected with *S. aureus*. (A) HIF 1α immunohistochemistry and (B) hematoxilin-eosin staining of renal mouse abscesses induced by intravenous infection with *S. aureus* seven days upon infection (following sections). HIF 1α positive nuclei are most abundant within the abscesses borders, some interstitial staining also occurs within the renal parenchyma. In addition, renal tubules in the vicinity of the abscess stain also positively (halfed arrow in B). A: arcuate artery, G: glomerulus, V: arcuate vein; arrowheads: margin of abscesses. Magnifications: (A) 250×, (B) 100×.

Finally, we employed a murine intravenous *S. aureus* infection model in which the *S. aureus* bacteremia results in kidney abscess formation [23]. After seven days upon infection, macroscopically visible kidney abscesses occurred. Kidneys were removed, fixed and embedded in paraffin. Serial sections were processed by hematoxylin-eosin (H&E)- and HIF-1 staining. Massive abscess formation was also detected in H&E staining (**Fig. 8B**) and HIF-1α positive nuclei were most abundant in the abscess-surrounding areas (**Fig. 8A**). HIF-1 activation was also detectable in the peritoneum of mice suffering from a *S. aureus*-peritonitis (see below).

Taken together, we conclude from the above described *in vitro*, *ex vivo* and *in vivo* data that HIF-1 activation is a general phenomenon in infections with human pathogenic bacteria, viruses, fungi and protozoae and that this phenomenon is most likely due to hypoxic signaling.

### Inhibition of HIF-1 by 17-DMAG increases survival of mice in a *S. aureus* peritonitis model

**Figure 9:**
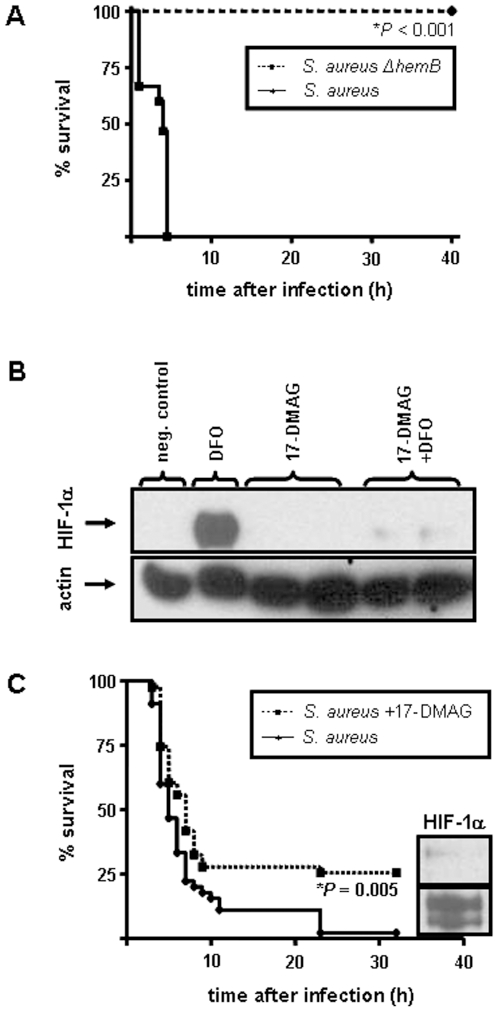
Role of HIF-1 in infections with *S. aureus* in a murine peritonitis model. (A) Survival of NMRI mice after intraperitoneal infection with *S. aureus* (wt; *S. aureus* 8325-4; n = 8) or *S. aureus* Δ*hemB* (SCV, *S. aureus erm*Ω*hemB* 8325-4; n = 8). Note the higher susceptibility of mice infected with *S. aureus* 8325-4 compared to the mice infected with *S. aureus* Δ*hemB*. *significant difference: P<0.001. (B) Inhibition of DFO induced HIF-1 activation in HeLa-229 cells by 17-DMAG. Cells were incubated with 17 DMAG (10 µmol/L) for 16 hours following induction of HIF 1 activation by the iron chelator DFO (200 µmol/L) for six hours. HIF 1α protein was analyzed in Western blots (loading control: actin). Negative control: uninfected cells. (C) Survival rate of NMRI mice after intraperitoneal infection with *S. aureus* (*S. aureus* 8325-4). One group of mice was treated 24 h and 16 h before infection with the HIF-1 inhibiting compound 17-DMAG (25 mg/g body weight). Note the higher survival rate of 17-DMAG-treated mice (n = 45) compared with control mice (n = 45). *significant difference: P = 0.005 (Kaplan-Meier analysis).

Mice are highly susceptible for a *S. aureus* peritonitis [24]. For this purpose, we infected mice intraperitoneally with *S. aureus* wild-type resulting in a 100% lethality of mice (n = 8). Infection with the related *S. aureus* SCV (*S. aureus ermBΩhemB*) strain (not activating HIF-1, see above) did not cause such a massive infection as all mice (n = 8) survived this infection (**Fig. 9A**).

As only *S. aureus* wild-type and not *S. aureus* SCV infections led to the activation of HIF-1 *in vitro* (see above), we finally analyzed the biological role of HIF-1 in this infection model. 17-DMAG is known to be a potent HIF-1 inhibitor [25]. Therefore we first established experiments elucidating the inhibition of HIF-1 activation by 17-DMAG in our experimental *in vitro* setting. In fact, addition of 17-DMAG prevented the activation of HIF-1 by DFO in a dose-dependent manner. A minimum concentration of 10 µmol/L 17-DMAG was effectual for inhibition of DFO-induced HIF-1 activation (**Fig. 9B**, not all data shown).

*In vivo*, the administration of 17-DMAG in fact prevented the peritoneal activation of HIF-1 during infection and this phenomenon was accompanied by a significant better survival of mice suffering from a *S. aureus* peritonitis. In detail, mice treated twice with 17-DMAG survived to a significantly higher percentage (10/38; 26%) compared to mice which did not receive 17-DMAG (1/38; 3%, **Fig. 9C**).

## Discussion

In this work, we demonstrate that HIF-1 activation in infections with human pathogenic microorganisms is a general phenomenon not restricted to certain pathogens. A robust and reliably detectable HIF-1 activation was shown (i) *ex vivo* in biopsies of patients suffering from skin infections, (ii) *in vitro* using cell culture infection models and (iii) *in vivo* using murine *S. aureus* infection models. Moreover, inhibition of bacterial induced HIF-1 activation resulted in higher survival rates in a *S. aureus* peritonitis model suggesting that the modulation of HIF-1-regulated pathways might influence the course of infections greatly.

In the past, hypoxia and iron-deprivation have been identified as the major conditions leading to HIF-1 activation [2], [3]. PHDs (mainly PHD-2) were shown to represent the molecular regulators of HIF-1 activity regulating ubiquitination and subsequent proteasomal degradation of the HIF-1α subunit [26]–[28]. According to these observations, we demonstrated earlier that HIF-1 activation by bacteria is either the result of cellular hypoxia following bacterial infections with e.g., *B. henselae* [7] or of iron-competition between bacteria and host cells in infections with certain *Enterobacteriaceae* [11]. From the herein described experiments it became obvious that HIF-1 activation by pathogens is a general phenomenon occurring in infections with bacteria, viruses, fungi and protozoae (see **Fig. 1** **–** [Fig pone-0011576-g002] [Fig pone-0011576-g003] **4**). This hypothesis is supported by various reports showing that HIF-1 activation occurs also in infections with, e.g., *Chlamydia pneumoniae* [29], *Helicobacter pylori* [30] and respiratory syncytial virus (RSV) [31]. Using bacterial and fungal infection models (HeLa-229 cells, NHEKs), we were able to link this HIF-1 activation with increased oxygen consumption [via (i) pimonidazole staining, (ii) direct quantification of oxygen partial pressure in cell culture supernatants and (iii) by overcoming cellular hypoxia using gas-permeable cell culture dishes, see **Fig. 2** **,** **3** **,** **7**] and strongly arguing for a role of PHD-2 and against transcriptional HIF-1 induction (excluded by quantification of HIF-1α mRNA, see **Fig. 4**, **Fig. S2**). Interestingly, in our experiments no increased HIF-2 activation was detectable when cells were infected with *P. aeruginosa*, *E. coli* and *S. aureus* (data not shown) and, until now, no reports are published indicating a role of HIF-2 in such settings.

Infections with *S. aureus* strains deficient in their aerobic metabolism [*S. aureus* Δ*hemB* [16], clinical *S. aureus* SCVs [21], [22]] did not influence the oxygen partial pressure in cell cultures and neither led to HIF-1 activation and oxygen consumption. HIF-1 activation was fully restored when infections with the respective parental wild-type or genetically complemented *S. aureus* strains were performed (**Fig 4** **,** **6** **,** **7**). Obviously, cell wall components of *S. aureus* (LTA, WTA, EAP) are not involved in HIF-1 activation (**Fig. 5**) arguing again for a crucial role of infection-triggered hypoxia. Interestingly, when using a murine peritonitis model [24], SCV-strains (deficient in their capacity of HIF-1 activation) turned out to be non-pathogenic (see **Fig. 9A**). Clinical SCVs are usually isolated from patients suffering from chronic infections (e.g., from lung infections due to cystic fibrosis or from osteomyelitis) [32]. It might be speculated that acute infections (e.g., with *S. aureus* wild-type) are linked to a rapid and strong HIF-1 activation whereas such activation is missing in chronic infections (e.g., with SCV bacteria) suggesting that the activation of HIF-1 in infections correlates directly with pathogenicity and the course of infection.

Although there is solid evidence for the impact of HIF-1 on the host response, the exact role of HIF-1 in infectious diseases is still largely unknown. The activation of HIF-1 by bacteria was demonstrated *in vitro* and *ex vivo* for the angiogenic bacterium *B. henselae* causing the vasculoproliferative disorders bacillary angiomatosis and peliosis hepatis [7], [10]. Here, *B. henselae* induces an angiogenic reprogramming of host cells via HIF-1. Infected cells secrete angiogenic compounds (e.g. VEGF) leading to the proliferation of endothelial cells [9], the assumed habitate of *B. henselae* [33], [34]. In contrast, for some members of the family of *Enterobacteriaceae* (*Y. enterocolitica*, *E. aerogenes* and *S. enterica* spp. *enterica*) an oxygen-independent HIF-1 activation was demonstrated. Here, bacterial siderophores (secreted by these *Enterobacteriaceae*) compete with host cells for iron affecting PHD2-activity and resulting in the activation of HIF-1 [11]. As mice with deletion of HIF-1α in the intestinal epithelium showed a significant higher susceptibility to orogastric *Y. enterocolitica* infections, bacterial HIF-1 activation appears to represent a host defense mechanism [11]. In fact, HIF-1 plays an important role in cellular host defense and innate immunity as HIF-1 is crucial for activation and stimulation of T cells by dendritic cells [35], regulates the bactericidal capacity of phagocytes [6] and the production of antimicrobial peptides (e.g., cathelicidin) [14]. Such defensins of the intestinal mucosa have been demonstrated to provide an antibacterial barrier to prevent infections with, e.g., *Enterobacteriaceae* [36]. Therefore, a lacking intestinal HIF-1 activity should finally result in a higher susceptibility for infections with, e.g., *Y. enterocolitica*. Similar mechanisms might also be operating in inflammatory bowel diseases (IBDs) where HIF-1 regulates the barrier function of the intestinal mucosa [37].

In contrast to the protective effect in intestinal infections, HIF-1 activation or induction of HIF-1 regulated genes seems to be detrimental in severe systemic infections. Strongly elevated VEGF serum levels were detected in septicaemic patients [e.g., suffering from a methicillin-resistant *S. aureus* (MRSA) infection] and in patients with severe meningitis [38]–[40]. HIF-1 activation was also observed in a lipopolysaccharide (LPS)-induced murine sepsis model [14]. Interestingly, functional blocking of serum VEGF by soluble VEGF-receptors decreased the mortality in a LPS-septicaemia model dramatically [15]. Concluding these observations and our results gained from the murine *S. aureus* peritonitis model (**Fig. 9A, C**) we suggest that an overwhelming HIF-1 activation (and subsequent VEGF secretion) is detrimental in severe infections. A mechanistic explanation for this hypothesis might be hidden in the vasculopermeability-increasing effects of VEGF which was originally described as vasculopermeability factor (VPF) [**41**]. In fact, VEGF-dependent vascular leakage was already demonstrated in patients suffering from septic shock [42].

Taken together, HIF-1 activation is obviously a general phenomenon in severe infectious diseases caused largely by hypoxia-dependent mechanisms. Our *ex vivo*, *in vitro* and *in vivo* data from infections with humanpathogenic bacteria, fungi, viruses and protozoae point towards a most important role of HIF-1 in the host defense against various pathogens. Extensions of these findings will determine the exact mode of HIF-1 activation by pathogens and the related biological effects in critically ill patients. The application of HIF-1 inhibiting compounds for the treatment of severe infections (such as peritonitis) remains to be elucidated in further studies.

## Materials and Methods

### Bacterial Strains

For *in vitro* experiments, the following microorganisms were used: the laboratory strains *S. aureus* ATCC 33592, *S. aureus* 8325-4, *S. aureus ermBΩhemB*, *S. aureus pCX19ΩhemB* [16] *S. aureus* SA113, *S. aureus* Newman, *S. aureus* ATCC 25923 and the clinical isolates *S. aureus* A22223 I (wt; patient isolate 1a), *S. aureus* A22223 II (clinical SCV; patient isolate 1b). *S. aureus* OM 1a (wt; patient isolate 2a) and *S. aureus* OM 1b (clinical SCV; patient isolate 2b). Additionally, the following strains defective in cell wall components were used: *S. aureus* SA113 *Δypf::ermB* (about 87% reduced lipoteichoic acid content), *S. aureus* SA113 *ΔtagO* (defective in producing wall teichoic acid) and *S. aureus* Newman mAH12 (defective in producing the extracellular adherence protein EAP). Other strains used for *in vitro* infection experiments were *Staphylococcus epidermidis* ATCC 12228, *Escherichia coli* ATCC 25922, *Pseudomonas aeruginosa* ATCC 27853, *Streptococcus agalactiae* SK 43 and *Candida albicans* ATCC 90028.

### Cell Culture and Infection Procedures of HeLa-229 cells and NHEKs

HeLa-229 cervix carcinoma cells were grown in VLE RPMI 1640 medium supplemented with 2 g/L NaHCO_3_ (Biochrom, Berlin, Germany), 10% heat-inactivated fetal calf serum (FCS; Sigma Aldrich, Taufkirchen, Germany), 1% L-glutamine (Gibco, Karlsruhe, Germany) and 10 mg/mL streptomycine and 100 U penicillin (Biochrom, Berlin, Germany). Normal Human Epidermal Keratinocytes (NHEKs) were grown in Keratinocyte Growth Medium 2 (PromoCell, Heidelberg, Germany) supplemented with the appropriate SupplementMix (supplied with Keratinocyte Growth Medium 2; PromoCell), 10 mg/mL streptomycine and 100 U penicillin (Biochrom).

For performing infection experiments, cells were detached with 0.05% Trypsin-EDTA (Gibco). After trypsinization of NHEKs, Trypsin-EDTA was neutralized by adding Trypsin Neutralizing Solution (PromoCell). Cells were seeded the day before infection in cell culture media without antibiotics (to allow bacterial growth). Infection experiments were performed in cell culture media without antibiotics and without FCS to avoid unspecific HIF-1 activation. The following pathogens were used: *S. aureus, S. epidermidis, S. pyogenes, C. albicans* [multiplicity of infection (MOI): 20, infection time: six hours], *S. agalactiae* (MOI: 200, infection time: six hours), *E. coli, P. aeruginosa* (MOI: 10, infection time: four hours). Uninfected cells were used as negative controls, desferrioxamine (DFO, 200 µmol/L, Sigma Aldrich) -treated cells or cells incubated under hypoxic conditions (1% oxygen; CO_2_ Incubator Innova CO-48; Eppendorf AG, Hamburg) were used as positive controls, respectively.

For inhibition of HIF-1 activation, HeLa-229 cells were treated with the heat shock protein (Hsp) 90-inhibitor 17-(dimethylaminoethylamino)-17-demethoxygeldanamycin (17-DMAG; LC laboratories, Woburn, MA, USA)[25]. Cells were incubated with 10 µmol/L 17-DMAG before stimulating HIF-1 activation by adding 200 µmol/L DFO.

### Detection of HIF-1 activation

For the detection of HIF-1 activation by immunoblotting, proteins from cell cultures were extracted as described [43], separated by 8% SDS-PAGE and blotted onto polyvinylidene difluoride (PVDF) membranes (Millipore, Schwalbach, Germany). Mouse anti-HIF-1α antibodies (Becton Dickinson, Heidelberg, Germany) were used as primary antibodies and horseradish peroxidase (HRP)-conjugated rabbit anti-mouse IgG antibodies (Dako, Hamburg, Germany) as secondary antibodies. Signals were visualized with the enhanced chemiluminescent (ECL)-reagent (PJK, Kleinbittersdorf, Germany). For loading control, mouse actin-specific antibodies (Sigma Aldrich) were used. In some experiments, mouse anti-human HIF-2α specific antibodies were used (NB 100–132; Novus Biologicals, Littleton, CO, USA).

To detect HIF-1α protein in infected cells, high-amplification immunohistochemistry was used as described previously [44]. HeLa-229 cells or NHEKs were seeded on coated glass slides (Superfrost Plus, Menzel, Germany) the day before infection. Four hours upon infection, cells were fixed with freshly prepared 3.75% paraformaldehyde (PFA; pH 7.4; dissolved in PBS) for 30 minutes and rinsed in PBS. Antigen retrieval was performed for 2 minutes in preheated target retrieval solution (Dako) using a pressure cooker. For detection of HIF-1α, monoclonal mouse anti-human HIF-1α (α67; Novus) and biotinylated secondary anti-mouse antibodies (Dako) were used. For signal amplification and visualisation, a catalyzed signal amplification system (CSA-Kit, Dako) based on a streptavidin-biotin-peroxidase reaction was used according to the manufacturers instructions. Between incubations, specimens were washed two to three times (50 mmol/L Tris-HCl, 300 mmol/L NaCl, 0.1% Tween-20, pH 7.6). DAB was used as chromogen for peroxidase-reaction. Untreated cells were used as negative controls.

Reporter assays were carried out using the dual-luciferase reporter assay (Promega, Mannheim, Germany). A pGL3-based hypoxia responsive element (HRE) plasmid containing four tandem HIF-1 enhancer sequences from the 3′-region of the erythropoietin gene upstream of the firefly luciferase gene [11] was cotransfected with a plasmid encoding a Renilla luciferase reporter construct for normalization [45]. Cells were transiently transfected using the ExGen 500 *in vitro* Transfection Reagent (Fermentas, St. Leon-Rot, Germany) and incubated for 24 hours under cell culture conditions. Cells were lysed four to six hours after infection with Passive-Lysis-Buffer (Promega). HIF-1-dependent luciferase activities normalized to Renilla activity were determined using a Packard TopCount NXT (BioScience, Groningen, Netherlands).

### RNA isolation, Reverse Transcription of Messenger RNA and Polymerase Chain Reaction

Total RNA of control and infected cells was isolated with the RNeasy Mini Kit (Qiagen, Hilden, Germany). To remove contaminating DNA from RNA preparations, the Turbo DNA-free™Kit (Applied Biosystems, Darmstadt, Germany) was used. Reverse transcription was carried out using SuperScript™ III Reverse Transcriptase (Invitrogen, Karlsruhe, Germany), RNaseOUT™ Recombinant Ribonuclease Inhibitor (Invitrogen), oligo-d (pT) 18 mRNA primers (New England Biolabs, Frankfurt am Main, Germany) and nucleotides (10 mmol/L dNTP Mix; Fermentas, St. Leon-Rot, Germany). HIF-1α, VEGF and actin gene expression was quantified using a Light Cycler 480 System (Roche Diagnostics, Mannheim, Germany). Primers and standard probes were obtained from LC Search (Heidelberg, Germany) as described earlier [11].

### Detection of Cellular Hypoxia and Oxygen Consumption

NHEKs and HeLa-229 cells were cultivated using conventional polystyrene dishes or special gas-permeable dishes (Lumox; Greiner Bio-One, Frickenhausen, Germany) with a hydrophilic tissue culture treated bottom membrane as described [46]. The pO_2_ (mmHg) was quantified in the medium of infected or uninfected control cells using the blood gas analyzer ABL-77, sensor cassettes and calibration packs (Radiometer, Willich, Germany). For internal control, cells were harvested in parallel four to six hours after infection, and whole cell extracts were prepared for the detection of HIF-1 activation by immunoblotting as described above.

Detection of hypoxia was also displayed by visualization of the hypoxic cell state using the hypoxia-sensitive marker pimonidazole hydrochloride (Natural Pharmacia International, Burlington, USA). Pimonidazole hydrochloride (which is a 2-nitroimidazole) forms adducts with thiol groups in proteins, peptides and amino acids in hypoxic cells (pO_2_ <10 mmHg) [47]. Here, 200 µmol/L pimonidazole hydrochloride (Hypoxyprobe-1) was added to HeLa-229 cells immediately before infection as described earlier [7]. After six hours of infection, cellular hypoxia was visualized using a primary monoclonal antibody IgG1 that detects protein adducts of pimonidazole in hypoxic cells (Hypoxyprobe-1Mab1; NPI, Burlington, USA) and secondary Cy3-conjugated goat anti-mouse IgG antibodies (Dianova, Hamburg, Germany). Visualization was performed using a Leica DM IRE2 confocal laser scanning microscope (CLSM; Leica Microsystems, Wetzlar, Germany).

### Intraperitoneal infection of mice with *S. aureus*

Female NMRI mice (33–35 g) were infected intraperitoneally with *S. aureus* 8325-4 or *S. aureus ermBΩhemB* 8325-4 (SCV) by injecting 0,3×10^7^ bacteria/g body weight in conditioned medium, as described before [24]. To study the role of HIF-1 *in vivo*, mice were inoculated additionally with the HIF-1 inhibitor 17-DMAG (25 mg/g body weight; application 24 and 16 hours before infection) as described previously [25]. Survival rates were measured half-hourly. Peritoneal HIF-1α activation was analyzed by immunoblotting of the shock-frozen peritoneal specimens using the Nuclear Extract Kit (Active Motif; Rixensart, Belgium), a mouse monoclonal anti-HIF-1α (H1α67) antibody (Novus Biologicals, Littleton, USA) and horseradish peroxidase (HRP)-conjugated rabbit anti-mouse IgG antibodies (Dako). Signals were visualized with the enhanced chemiluminescent (ECL)-reagent (PJK). Normalization of the samples used for immunoblotting was performed using a Pierce protein quantification assay (BCA Protein Assay; Thermo Fisher Scientific, Bonn, Germany).

### Intravenous infection of mice with *S. aureus*

Mouse infection experiments were done in accordance with the animal experiment proposal (H2/06, Tuebingen, Germany, approved by the Regierungspräsidium Tübingen, Germany). The Institutional Animal Care and Use Committee approved this protocol. Female NMRI mice (33–35 g) were infected intravenously with *S. aureus* 8325-4 (0,3×10^6^ bacteria/g body weight) resuspended in 200 µl conditioned medium as described previously [23], [24]. Seven days after infection, mice were euthanized and kidneys were taken out when a macroscopically visible abscess formation had occurred. Organ fixation was performed using 3.75% PBS-buffered (pH 7.4) paraformaldehyde (PFA).

### Selection of patient samples

Formalin-fixed, paraffin-embbed diagnostic skin biposies were collected from the files of the Department of Dermatology, University of Tuebingen. Only samples for which a microbiological diagnosis of the underlying infection was made in parallel were included in the study. Microbiological pathogen identification was performed using standard microbiological techniques [48].

### Histology and HIF-1 immunohistochemistry of patient and murine samples

For murine kidney samples, serial sections (thickness of the sections: 2 µm) were done and processed by hematoxylin-eosin and HIF-1α staining. Human samples were processed directly from paraffin-embedded blocks.

Paraffin sections (2 µm) were dewaxed in xylene, rehydrated in a series of ethanol washes, and placed in distilled water before staining procedures. Slides were coated with 3-aminopropyl-tri-ethoxysylane. For detection of HIF-1α isoforms, a monoclonal mouse anti-human HIF-1α antibody was used for patient samples (α67; Novus Biologicals) and polyclonal rabbit anti-mouse HIF-1α antibodies were used for murine samples (PM16, obtained from a rabbit immunized with a peptide containing amino acids 553 to 669 of mouse HIF-1α; friendly gift from Patrick Maxwell, Imperial Hospital, London, UK). Detection of bound antibodies was performed by using biotinylated secondary anti-mouse (patient samples) or anti-rabbit (murine samples) antibodies and a catalyzed signal amplification system (Dako) based on the streptavidin-biotin-peroxidase reaction, according to the instructions provided by the manufacturer. Antigen retrieval was performed for 90 s in preheated target retrieval solution (Dako) using a pressure cooker. All incubations were performed in a humidified chamber. Between incubations, specimens were washed two to four times in buffer (50 mmol/L Tris-HCl, 300 mmol/L NaCl, 0.1% Tween-20, pH 7.6). For counterstaining, hematoxylin-eosin staining of the corresponding section was done using standard laboratory procedures.

### Statistical analysis

For statistical analysis, the unpaired, two-tailed Student *t* test was used. Analysis of the survival rates of mice was performed by Kaplan-Meier analysis using the GraphPad Prism Software (GraphPad Software Inc., La Jolla, USA). For all assays, a value of *P*<0.05 was considered statistically significant.

## Supporting Information

Figure S1 Induction of HIF-1 activation in HeLa-229 cells by bacterial pathogens. Hela-229 cells were seeded on glass slides infected with *P. aeruginosa* ATCC 27853 or *C. albicans* ATCC 90028. HIF-1 activation was detected by nuclear accumulation of HIF-1α via immunohistochemistry six hours upon infection. Negative control: uninfected cells; positive control: DFO (200 µmol/L). Scale bar: 20 µm.
(1.47 MB TIF)

Figure S2 Induction of HIF-1-dependent gene programming by bacterial pathogens in HeLa-229 cells. Transcriptional analysis of (A) VEGF or (B) HIF 1α upon infection. Total mRNA was prepared four to six hours upon infection, transcribed into cDNA, and mRNA was quantified by real-time Light-Cycler-PCR (ratio: VEGF/actin or HIF 1α/actin transcripts; triplicate means given). * significant difference to control cells (P<0.05).
(0.82 MB TIF)
