# Supplementary figures and images for: Activation of Hypoxia Inducible Factor 1 Is a General Phenomenon in Infections with Human Pathogens
Source: PLoS One. 2010 Jul 14;5(7):e11576. doi: 10.1371/journal.pone.0011576 (PMC2904385; doi:10.1371/journal.pone.0011576)

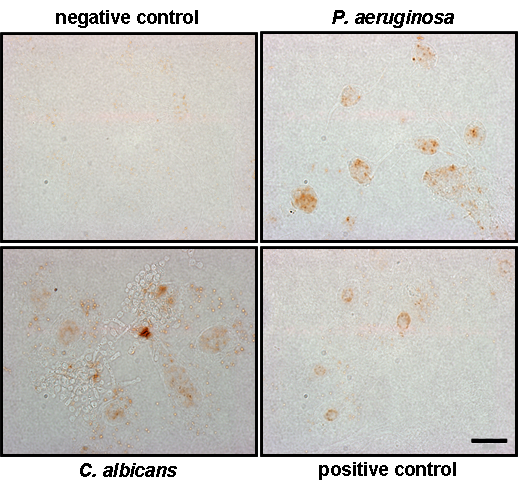

Supplement: Figure S1 — Induction of HIF-1 activation in HeLa-229 cells by bacterial pathogens. Hela-229 cells were seeded on glass slides infected with P. aeruginosa ATCC 27853 or C. albicans ATCC 90028. HIF-1 activation was detected by nuclear accumulation of HIF-1α via immunohistochemistry six hours upon infection. Negative control: uninfected cells; positive control: DFO (200 µmol/L). Scale bar: 20 µm. (1.47 MB TIF) [file pone.0011576.s001.tif]

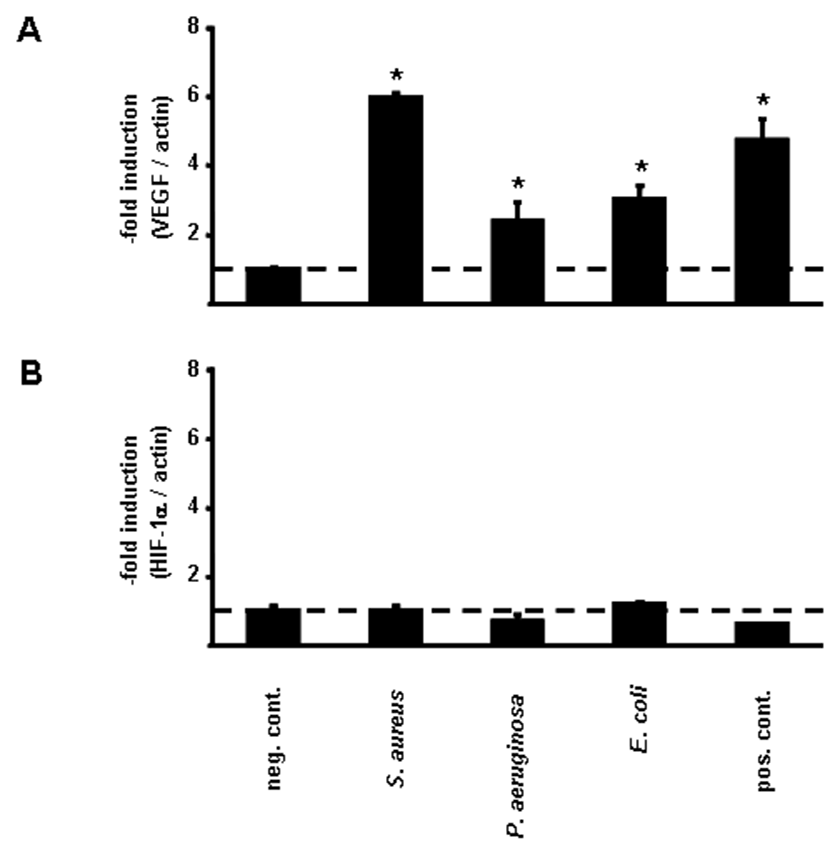

Supplement: Figure S2 — Induction of HIF-1-dependent gene programming by bacterial pathogens in HeLa-229 cells. Transcriptional analysis of (A) VEGF or (B) HIF 1α upon infection. Total mRNA was prepared four to six hours upon infection, transcribed into cDNA, and mRNA was quantified by real-time Light-Cycler-PCR (ratio: VEGF/actin or HIF 1α/actin transcripts; triplicate means given). * significant difference to control cells (P<0.05). (0.82 MB TIF) [file pone.0011576.s002.tif]
